# Framework for Patient and Informal Caregiver Participation in Research (PAICPAIR): Part 1

**DOI:** 10.1097/ANS.0000000000000289

**Published:** 2019-11-04

**Authors:** Katrine Staats, Ellen Karine Grov, Bettina Husebø, Oscar Tranvåg

**Affiliations:** Department of Global Public Health and Primary Care, Centre for Elderly and Nursing Home Medicine, University of Bergen, Norway (Ms Staats and Drs Husebø and Tranvåg); Institute of Nursing and Health Promotion, Oslo Metropolitan University, Norway (Dr Grov); Norwegian National Advisory Unit on Women's Health, Oslo University Hospital, Rikshospitalet, Norway (Dr Tranvåg); and Faculty of Health and Social Sciences, Western Norway University of Applied Sciences, Norway (Dr Tranvåg).

**Keywords:** home health care, human dignity, palliative care, practice guidelines, research, research ethics, user involvement

## Abstract

The term *user involvement* is frequently applied in research. Frameworks for patient and informal caregiver participation as coresearchers in studies concerning patients with life-threatening illness are however sparse. The PhD project *Dying With Dignity*—*Dignity-Preserving Care for Older Women Living at Home With Incurable Cancer* has implemented a thorough cooperation with patients and informal caregivers from the early stages of the research process. A framework for *Pa*tient and *I*nformal *C*aregiver *Pa*rticipation *I*n *R*esearch (PAICPAIR) is suggested—creating a stronger foundation for democracy, equality, and research quality by also promoting active participation among vulnerable people experiencing incurable, life-threatening illness, as coresearchers.

FROM THE VERY START, the research project involving vulnerable human beings, like patients experiencing incurable life-threatening illness, carries with it ethical challenges and considerations toward establishing user involvement. Simultaneously, it is ethically challenging *not* to include these patients and caregivers in a research project solely based on vulnerability. The concept *user involvement* has been discussed and integrated in research for a long time. However, we argue that *user involvement* is a vague abstraction of vital research processes concerning how to include both patient and informal caregivers as coresearchers—a perspective found sparsely in literature. Consequently, in our view, the *patient*, not *user*, should be in focus as well as the *informal caregiver*. An *informal caregiver* is a family caregiver or another person close to the patient who plays an important role in his or her own right, other than patient, or “user”—in accordance with the Norwegian legal system.^1^ The purpose of this article is to formulate a framework for patient and informal caregiver participation in research—replacing the old focus of *“them” as research objects*, with focus on *“us” as fellow researchers*. The framework “Patient and Informal Caregiver Participation in Research” (PAICPAIR) is founded upon perspectives, ideas, and experiences made during the PhD project *Dying With Dignity*—*Dignity-Preserving Care for Older Women Living at Home With Incurable Cancer* (*Dying With Dignity* project) to be presented here. But first, and importantly, in some countries and cultures dying with dignity has been discussed in relation to assisted suicide of seriously ill patients suffering from incurable diseases.^2^ This present study is not based on this perspective, but on the ontology, caring science, and caritative caring theory developed by Katie Eriksson^3^–^5^—emphasizing preservation of the absolute dignity of all humans, the respect for human wholeness consisting of body, soul, and spirit, and the creation of room for life and living.

Introducing the PAICPAIR framework, we will first account for the Norwegian concept of user involvement within the health care system, to show that there is an important connection between “user involvement” in health care practice and health care research. Thereafter, a historical perspective of the usage and development of this concept within the research context will be given.

**Statements of Significance**

**What is known or assumed to be true about this topic:**

Research on vulnerable human beings implies ethical challenges and considerations. However, we believe it is unethical not to include patients and their informal caregivers in research projects solely based on vulnerability. Little attention is paid to how vulnerable people, living with incurable, life-threatening illness and their informal caregiver actively can participate as coresearchers. Hence, there is a need for developing a research framework—creating a stronger foundation for democracy, equality, and research quality through active participation of patient and informal caregiver representatives experiencing incurable, life-threatening illness, as coresearchers.

**What this article adds:**

This article is the first of 2 presenting a framework for Patient and Informal Caregiver Participation in Research (PAICPAIR). The framework is founded on learning from research collaboration with a patient and 2 informal caregivers experiencing incurable, life-threatening illness—participating as our coresearchers in the *Dying With Dignity* project. We describe and discuss crucial aspects of the first part of this research collaboration—how we constituted and developed a formal research collaboration, as well as how we supported and empowered the coresearchers during the initial stages developing study aim, interview guides and letters of information.

## USER INVOLVEMENT—IN HEALTH CARE AND RESEARCH

### A legal right within the municipality health care system in Norway

In the Norwegian health care system, user involvement is organized on 3 levels, all regulated by different legal systems. First, on the individual level, we find patients who participate in decisions concerning their care and medical treatment. Their rights are included in the Norwegian *Health and Rights Act.*^1^ This law regulates, among others, the right for the patient to receive relevant information as well as active participation in discussions and decisions concerning eventual treatment. Second, on the service level, patients and informal caregivers shall be included in user involvement processes. Their rights are regulated by the Norwegian *Regulations on Management and Quality Improvement in the Health and Care Services*.^6^ Their experiences shall be utilized toward health care service improvements within the municipality. Third, on the system level, the municipality is obligated to listen to, and include representatives of user groups in developing health care services, regulated by the *Act relating to Municipal Health and care Services.*^7^ In this article, we argue that research studies, like the *Dying With Dignity* project, are in accordance with the legal obligations of promoting patient and informal caregiver inclusion in knowledge development—relevant for the individual level, service level, and system level of municipal health care. To better understand how “user involvement in research” has become a vital principle, a historical view of the development, as well as knowledge of the various concepts used to communicate this principle, will here be presented.

### Historical view of user involvement in research

The history of user involvement in research began in the 1960s in the United Kingdom. People with disabilities struggled for more control over the production of research knowledge, lobbying for full recognition as worthy human beings.^8^ From the 1980s, user involvement in research was perceived as a political measure to increase research quality on the municipal level. However, user involvement within the research context in Norway had its breakthrough in the 1990s. *The White paper nr. 41*,^9^ concerning health politics toward year 2000, demanded increased user involvement in research, recognition of participant experience as valuable knowledge, boosting democratization within health care services. In recent years, the patient's perspective has been increasingly recognized as an important indication of health care quality and is frequently cited in international health policy documents.^10^ In Norway user involvement is established as a core value and a designated health policy goal.^11^

#### A variety of concepts describing user involvement

During the last 2 decades, *user involvement* in research has received greater recognition among research professionals. A researcher's perspectives are however anchored in his or her ontological foundation. Therefore, like ripples on water, words and terms describing the researcher's point of view follows his or her ontological perspective of the world. Not surprisingly, a review of the literature reveals a variety of concepts utilized when describing the inclusion of *users* in a given research project. A minor literature review including 30 studies was conducted to identify how the “user” concept and concept with similar meaning applying other words are utilized—and create a basis of origin. The UK and the United States represented, respectively, 15 and 7 of the 30 studies, whereas Australia, Canada, the Netherlands, and Spain were responsible for the remaining 8 studies. There is a striking emphasis visible in the UK's agenda on “user involvement” in research, while the Scandinavian countries are conspicuous by its absence so far. The concept *user involvement* is of course widely used, for example Staniszewska et al^12^ and Wright et al.^13^ Related terms include *service user involvement*,^14^,^15^ *user-led research*,^16^ and *user-controlled research.*^17^ *Consumer involvement* is also widely used, for example, in the work of Hanley et al^18^ and Kreis et al.^19^ Associated terms such as *including consumers*,^20^ *consumer participation*,^21^ *consumer and community participation*,^22^ and *consumer-driven*^23^ are also found in the research literature. In some studies, *collaborative partnerships*,^24^ *citizens' juries*,^25^ and *public involvement*^26^ are chosen, while others make use of expressions like *public participation*,^27^ *community participation*,^28^ or *community researchers.*^29^ Some researchers also use phases like *engage with stakeholders*^30^ and *involving people affected (by illness).*^31^ While Whitley and Goldman^32^ prefer the term *client involvement*, Sacristán et al^33^ promote the expression *patient involvement.* Like Sacristán et al, several researchers employ the word *patient* in their concept construction, for example, Nierse et al^34^ with *patient research partners*, Marsden and Bradburn^35^ applying *patient and clinician collaboration*, Harrison et al^36^ employing *patient stakeholder engagement*, and Shippee et al^37^ emphasizing *patient and service user engagement.* Furthermore, while Staniszewska et al^38^ promote *patient and public involvement* as their concept, Abma and Broerse^39^ advocate *patient participation*, and Price et al^40^ encourage the use of *public and patient participation.* Jones et al^41^ recently published an article using the term *patient and carer participation.* The latter study corresponds with central ideas and perspectives our research group identified and addressed in the *Dying With Dignity* project. The studies above give little attention to discussions concerning the possibilities and experience of vulnerable people with life-threatening illness and their informal caregiver—participating in research. Hence, there is a need for developing a research framework—creating a stronger foundation for democracy, equality, and research quality through active participation, also including vulnerable people like patients experiencing incurable, life-threatening illness, as coresearchers. The purpose of this article is, objective, to present and discuss the framework “*Patient and Informal Caregiver Participation in Research*” (PAICPAIR)—founded upon our perspectives, ideas, and experiences during the *Dying With Dignity* project.

## INTRODUCING THE PAICPAIR FRAMEWORK

### Ontological foundation

As caring theory and caring science^3^,^4^ places patient and informal caregivers in the center of care interventions, we argue that *patient and informal caregiver participation* is a term that should be employed in research investigating various aspects of living with illness, both as patient and informal caregiver. We have observed how the terms *user* and *consumer*, both concepts anchored within the perspectives of New Public Management (NPM) ideology, weaken the focus as well as the understanding of patient and informal caregiver perspectives. NPM is an approach to run public services, such as health care organizations, more “businesslike” and to improve efficiency, which is widely criticized.^42^ The Norwegian *Health and Rights Act*^1^ clearly states: *A user* is not synonym with *a patient*. In other words, *patient* is not a synonym for *user*. While a patient is a person receiving health-related treatment and care, a user is a person gaining non-health-related services, for example a person being supported by the municipality due to difficulties in cleaning his/her house or buying groceries. Additionally, we believe promoting active *participation*, instead of the more vague term *involvement*, creates an even stronger foundation for democracy and equality, empowering patients and informal caregivers as coresearchers, together with established professional researchers. As underscored by caring scientist and theorist Katie Eriksson,^3^ every age has its questions that most profoundly reflect the ripeness of the science that poses the questions and the underlying conception of reality. The depth of the questions is determined by the basic concepts we have at our command. Consistent with Eriksson,^4^ we argue that refining research concepts is a core task for science. Equally important is the need for more humanistic-oriented thinking and conceptualization. On this basis, we suggest aligning people who experience incurable life-threatening illness at the forefront—also as they participate in developing and conducting research.

### Objective

A main objective of the *Dying With Dignity* project is the development of a framework for patient and informal caregiver participation in research, also including seriously ill people and their informal caregivers, as coresearchers. The World Health Organization^43^ states that dignity-preserving care increases quality of life among patients with life-threatening diseases while the Norwegian health and care authorities aim to secure all citizens the right to die in a dignified manner.^44^ However, knowledge concerning factors preserving a dignified death at home is sparse. Studies show the majority of older people desire to live and die in the familiarity of their own home.^45^,^46^ Additionally, the Norwegian Research Council encourages increased research on gender-specific aspects related to women's health and disease.^47^,^48^ However, the questions remains: What is dignity? Who shall define dignity, and for whom? And more specifically, how can we study the basis for dignity and loss of dignity among older women living with life-threatening illness at home? The knowledge on this is limited and therefore the aim of the *Dying With Dignity* project is to identify and document factors preserving dignity as well as leading to dignity loss among older women living with incurable cancer at home. For us, it has been important to include representatives of these women presently experiencing similar life situation as our coresearchers, establishing a formal research collaboration with female patients with incurable cancer living at home as well as informal caregiver representatives, to access their experience-based knowledge in all stages of the research process. Our patient coresearchers have the similar inclusion criteria as the recruited participants in the *Dying With Dignity* project: female, 65 years or older, diagnosed with cancer in a palliative phase, living at home, and receiving support from municipality services. The informal caregivers being our coresearchers are, or have previously been, a family caregiver of a female patient, 65 years or older, living at home with cancer in palliative phase. However, the coresearchers are excluded as participants in interviews and observations, to avoid double roles. Patient and informal caregiver participation in research is important because of a growing awareness of its relevance and positive impact on the quality of research. When bringing the conceptualization “patient and informal caregiver participation in research” to the forefront, also including people experiencing serious illness, we aim to stimulate and motivate other global (north) countries to look at the health care policy goals, which have emerged from this shared governance, enhancing democracy, shared power, and knowledge development through new collaborative relationships within the research context.

### Constituting and developing a formal research collaboration

In line with Hoddinott et al^49^ and Blackburn et al^50^ on incorporating patients and public perspectives in research, we acknowledge patients and informal caregiver representatives as experts within the subject under investigation, making their contributions and unique competence of great value to all phases of the research process. This consists, among others, of formulating the study's aim, participation in developing information letters and interview guide, as well as reviewing analyzes and article drafts. In accordance with the legal systems mentioned earlier, the *Dying With Dignity* project has established systems for gathering both patient and informal caregiver perspectives. One patient representative and one representative for informal caregivers are seated on the projects' advisory board, while one informal caregiver is seated within the steering group. Regularly scheduled planning sessions plus systematic follow-up phone calls help ensure the project receives adequate consideration in planning as well as the completion of various study processes. This coresearch process encourages collaboration among established researchers together with patient and informal caregiver representatives, as they combine their efforts toward developing the study of crucial aspects in promoting a worthy, dignified death, for older women with incurable, life-threatening cancer, living at home. During this initial phase of research collaboration, also called a codesign process and the first step in a broader coresearch process, 2 main questions have been addressed: *(1) How can we organize, in both a practical and an ethical manner, patient and informal caregiver participation in this study? (2) How can patient and informal caregiver participation improve research tools and research outcomes?*

In the following text, we reflect upon and discuss these vital questions.

One important perspective has been to change focus from research *involving users* to research *collaboration together with* patient and informal caregiver representatives. Distancing ourselves from traditional research perspectives, *we* versus *them*, helped us to overcome these limiting standpoints and include patients and informal caregivers as co-researchers in a new perspective: *us*.^51^ In this current project, we began early to contact patient organizations with the aim of establishing their participation in our steering group as well as on our advisory board. As previously recommended, Daveson and collegues^52^ verify the importance of early inclusion of participant representatives in the research process. In their study, early and flexible participation seemed a requirement for success, as both patient and researcher highlighted early cooperation a necessity to ensure real and meaningful impact on the research process.^52^ In our *Dying With Dignity* project, patient and informal caregiver representatives were recruited through the Norwegian Cancer Society early in the planning phase—to our advisory board as equal to representatives with health care professional backgrounds. Our advisory board members include a current patient, a former informal caregiver, as well as an oncology nurse and a general practitioner from the municipality (Figure). In the initial phase, correspondence occurred mostly by email between patient/informal caregiver board members due to illness-related challenges and practical reasons. The advisory board has given their recommendations on the project plan, interview guide, and the letters of information for our recruitment processes. The projects' steering group consists of a project manager (PhD candidate) and 3 professional researchers (and PhD supervisors), a former informal caregiver to an older woman with incurable cancer, a senior researcher at the National Advisory Unit on Women's Health, Oslo University Hospital (Rikshospitalet), and a senior adviser for Women Health and research at Norwegian Women's Public Health Association (Figure). All have competence in oncology diseases and health care issues, including insight into the needs and current life situation of older women. Both informal caregivers participating as coresearchers have cared for their mothers, thus conforming to inclusion criteria. In this initial phase of the project, the steering group discussed feedback given by the advisory board and passed resolutions concerning the mentioned research documents. In addition, both groups maintain an ongoing evaluation of the overall project process.

**Figure.**
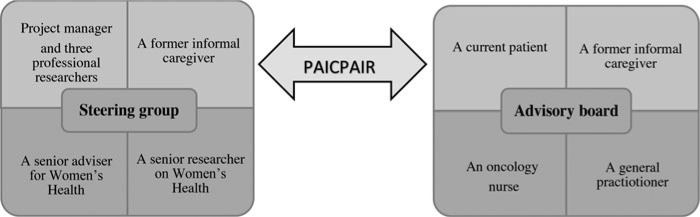
Patient and Informal Caregiver Participation in Research (PAICPAIR).

There are several important questions to address in this process: How can patient and informal caregiver representatives participate in research? Do we merely want them to read our project protocol and propose suggestions, which professional researchers determine whether to include or not? Alternatively, do we want patients and informal caregivers to participate as coresearchers and perhaps even coauthors? Just as the responsible therapist cannot disfranchise his or her professional obligations,^53^ neither can the professional researcher renounce the responsibility in determining the answers to these crucial questions. As previously mentioned, patient and informal caregivers within the *Dying With Dignity* project have given their recommendations on several documents, as well as participating in meetings with the project manager. Then all feedback from the coresearchers has been discussed thoroughly by the research team, and implemented within the preliminary results of the research project. The patient and informal caregivers will be asked to take the role as coauthors of *Dying With Dignity* project publications as long as this is not perceived as an undue burden in their present life situation.

### Establishing a collaboration of mutual learning

We will now look deeper at the advantages as well as challenging aspects of PAICPAIR in practice. It is arguable that patient and informal caregiver participation can strengthen data quality. In qualitative interviews, for example, when carried out by a patient and not a professional, the informant may experience the power relationship more equal, and research results may vary from interviews carried out by traditional researchers, increasing relevance for health workers and health care users.^54^ Nevertheless, and importantly, there are challenges, as well as limitations to this patient and caregiver influence. Forbat and Hubbard^55^ found that user involvement, to use their term, might lead to contrary rather than collaborative accounts. In their study, former informal caregivers were included as coresearchers to conduct interviews with current informal caregivers concerning their experiences. Even with preinterview training, conversational analysis identified evidence of interactional difficulties across the data. Coresearchers often referred to their own experiences as informal caregivers, frequently changing topics, sometimes missing opportunities for further disclosure.^54^ This phenomenon may be the results of difficulties in separating roles as current informal caregivers versus coresearchers, as well as a lack of awareness of their own preunderstanding, factors generally utilized constructively in interview settings in traditional research. Structural practices and frameworks are important to implement sustainable relationships when professional researchers, patients, and informal caregivers join forces in research projects. As part of professionalism, we argue that the professional researcher, during the entire research process, pays close attention to the following questions: What does my coresearcher need to conduct her/his work? How can I contribute to support and strengthen the coresearcher's foundation for performing her/his coresearcher tasks? And last, but not least, how can I as a professional researcher learn from my coresearcher(s)? It is crucial to bear in mind that the professional researchers can gain new substantial insights, leading the research process into fruitful pathways from the rich understanding of those experiencing illness and suffering. In our *Dying With Dignity* project, we have been inspired by INVOLVE guidelines, a well-known framework for patient and public participation in research developed by National Institute for Health Research in Great Britain.^51^ These guidelines emphasize research identification, prioritizing, design, administration, and dissemination. Here, training aims at helping representatives of the public receiving health care services toward developing knowledge, skills, and experience preparatory to their role as coresearchers. Their contributions in the commissioning stage gave the project a broader perspective toward considering issues of importance from their unique perspectives. Once training was funded, they gave relevant feedback on the project plan. They also helped develop plain language, patient- and informal caregiver-friendly, written information. These are crucial recommendations formulated in the INVOLVE guidelines concerning project design and management.^51^ Based on our experience from the *Dying With Dignity* project, early participation of patient and informal caregiver representatives helps build and strengthen the relevance, quality, and ethical sensitivity by including coresearchers experiencing incurable, life-threatening illness. Most important for our study, a prerequisite for patient and informal caregiver representatives has been empowerment toward their new coresearcher roles.

### Empowerment—meeting the coresearchers' needs

Our *Dying With Dignity* project concludes that the coresearchers, 1 older woman living at home with incurable cancer and 2 informal caregivers, are all highly vulnerable in their present life situation. Our obligation of moral sensitivity and ethical considerations in all parts of the research process is therefore evident. Participation in research may be experienced as meaningful and supportive of patients' and informal caregivers' current life situation. On the other hand, persons living with serious, potentially disabling or life-threatening diseases can be highly vulnerable.^56^ As the inclusion criteria for patient and informal caregiver indicate, acting as coresearcher may potentially be a burdensome and negative experience for some due to current life situations, for example, experiencing emotional strain after cooperating in developing an interview guide for research among women with a similar illness and suffering as oneself. In our *Dying With Dignity* project, we paid attention to these potential emotional consequences and offered support on several levels. As an example, the project manager carried out home visits to the patient in the advisory board and followed up with monthly phone calls. Likewise, a coresearcher situated in an interview setting, interviewing another person having a comparable and life-threatening life situation, can obviously be an emotional burden as they deal with poignant experiences^57^—highlighting how ethical implication is a crucial part of patient and informal caregiver participation in research. Due to our experiences with patient and informal caregivers' vulnerability in their present life situations, we have thoroughly discussed possible implications within the research team. We concluded that the patient and informal caregivers must be protected from potential burdens when acting as coresearchers in the interview setting, and ought therefore not participate as coresearcher in this step of the project. We humbly emphasized the exploration of their needs as well as how best to inform them concerning their role in the process. We strove to give adequate information concerning all sides of the research process, also emphasizing their right to exit the project at any time prior to publication. One coresearcher, a patient still receiving palliative treatment in a late stage of her illness, felt emotionally burdened while reading related documents and coping with the responsibility of being a member of the advisory board. As a research team, we therefore offered emotional support and additional time, enabling her to choose to go forward and complete her work as coresearcher. Wright et al^58^ suggest emotional support be provided for each co-researcher experiencing distress related to his or her new coresearcher role. Anchored in the *Dialogue Model*,^59^ we emphasized supportive follow-up phone calls to meet the needs of the woman just mentioned in her role as our coresearcher. The *Dialogue Model* is an approach aimed toward strengthening relationships, for example, between coresearchers like herself and our professional research team. Within the academic context, emphasis is placed on including and facilitating patient participation in research by striving for a foundation of equal partnership, strong relationships, and shared goals.^59^ Due to the unpredictability inherent with cancer among our coresearcher, there was a chance of reduced physical condition as they received palliative care. Additionally, previous informal caregiver representatives have shown us how their life situation can make it hard for them to find time to attend research group meetings. We chose therefore to offer our coresearchers alternative means for receiving information, such as home visits instead of reading several documents via email. Alternatively, ethical considerations were emphasized toward preserving their integrity and dignity by presenting documents verbally, followed by a discussion on their own terms.

As we evaluate the PAICPAIR process thus far, we discovered various needs among patients and informal representative co-researchers. One is their vulnerability and unpredictability as they struggle day by day. Based on these realizations, we found it necessary to broaden the advisory board by approaching 2 new patients and informal caregiver organizations. At this point, we hope to invite an additional patient, an informal caregiver, and an oncology nurse to the advisory board. By engaging additional patient and informal caregiver representatives, we aim to create a structure where the coresearchers' responsibilities and duties are shared among a larger group of people—ensuring that work as coresearchers does not become an additional burden in their already demanding life situation, while strengthening the PAICPAIR approach. Continual evaluation of our success in recruiting and sustaining patient and informal caregiver representatives will be emphasized throughout the project to increase our knowledge of how these crucial aspects can be safeguarded within the PAICPAIR approach. (Our experiences and lessons learn from these continuous evaluations will be thoroughly described in a later article of the PAICPAIR framework part 2.)

For coresearchers participating in the data collection process, extensive training must also be provided.^58^ The focus of such training should be a basic understanding of the research process and outcome measurements.^60^,^61^ Simultaneously, it is important to tailor training toward individual preferences and needs. Training sessions should be short, held over successive days, even offered “online” for convenient patient and coresearcher viewing.^61^ In their model, Daveson and colleagues^52^ show how diverse virtual and face-to-face methods can be used to ensure flexibility in patient and public involvement in research. In the *Dying With Dignity* project, the first author has prioritized personal meetings with all members of both steering group and advisory board, except for one former informal caregiver who presently feels too emotionally exhausted to talk about personal experiences due to her mother's recent death. However, based on our email communication, she found it expedient to write down her thoughts and experiences, delivering them by email. Our personal meetings with the coresearchers were fruitful, bringing clarification on various issues, such as expectations on the amount of time spent on the project, as well as providing support to help reduce the sense of insecurity in their new role as well as to lower their self-imposed pressure to perform at such an advanced level.

Reflecting on the realities and values of patient and informal caregivers as coresearchers, Banks and Brydon-Miller^62^ emphasize how ethical issues should be supplemented by research guidelines, to encompass matters such as harms, benefits, and responsibilities experienced by the average person. The necessity of researcher communication skills and relationship building toward gaining coresearchers' confidence, meeting them with honesty, faith, and care, rather than merely moving forward with the research project, is of great importance. Therefore, a consequence of omitting patient and informal caregivers as coresearcher at this research step may be a limitation to our study. In our project, when deciding an omission of patients and informal caregivers in certain steps of the research process, we make a division of power visual, helping us uphold moral sensitivity and ethical standards on how and when coauthors shall and shall not participate actively. An example of this in this present study is our decision not to include coresearchers in performing interviews with people experiencing similar life-threatening, incurable illness as themselves.

### Strengthening study validity

Validity is essential for research trustworthiness, integrity, and quality.^63^ It is therefore timely to ask at this stage of our framework development: Has PAICPAIR helped thus far to strengthen validity of the *Dying With Dignity* project? Based on our evaluations, the answer is yes. Our research team has received substantial, constructive feedback from our advisory board as well as considerable guidance from our steering group. After discussing these comments and recommendations, we decided to implement most of the patient and informal caregiver responses in our research strategy. For example, a former informal caregiver in our steering group, who recently had provided substantial homecare for her mother, added several important improvements to the interview guide. One example of this concerned the implementation of questions concerning degrading experiences among patients confined to bed and incontinence. Additionally, the patient and informal caregiver representatives encouraged addressing sensitive issues relating to intimate care during the first home nurse visit, including clarification of preferred terminology and communication procedures. Thus, as in our case, patients and informal caregivers as co-researchers may bring nuances and contextual perspectives from their personal experience into the study—which we as professional researchers have paid little or no attention to at all. As a result, these and other improvements in the quality of our data collection tools, as well as the validity of the collected empirical data and outcomes, will likely increase. We argue that PAICPAIR, early inclusion of patient and informal caregiver representatives with illness-related experiences in designing our research project, helped strengthen study validity. As members of our steering group and advisory board, they became the decision-makers concerning which research questions were most relevant to investigate.

## CONCLUSIVE CONSIDERATIONS AND FURTHER RESEARCH

In conclusion, utilizing PAICPAIR as inspired by the INVOLVE guidelines has improved research quality through patient and informal caregiver inclusion, training, and support. Ethical considerations, study advantages, and challenges, as well as attention to patient and informal caregiver representatives, have been crucial throughout the research process. A strong working relationship among professional researchers together with our patient and informal caregiver co-researchers, combined with continual awareness of their potential vulnerability while consciously placing *us* at the center of our research project, has been a vital foundation throughout the PAICPAIR process thus far. This includes constructive feedback by our coresearchers, helping to enhance project quality and validity. The unique strengths of this project include clarity of attitude, willingness and engagement of our patient and informal caregiver representatives, and the introduction of PAICPAIR as a means of obtaining increased understanding—developed as the main objective of this study investigating crucial factors preserving dignity or leading to loss of dignity among older women living with incurable, life-threatening illness at home.

This is the first of 2 articles presenting and describing a framework for Patient and Informal Caregiver Participation in Research (PAICPAIR framework part 1). This framework is based on our learning experiences while conducting the *Dying With Dignity* project in collaboration with patient and informal caregivers experiencing incurable, life-threatening illness, as coauthors. During the first part of this research endeavor, we describe and discuss crucial aspects toward the constitution and development of a formal research collaboration, and how to empower and support our coresearchers during the initial process stages, developing the study's aim, interview guides, and letters of information.

Learning experiences from the second and conclusive part of the PAICPAIR process will be published in a later article (PAICPAIR framework part 2)—a description and discussion of crucial themes relating to the analysis process, development of results, how our combined research team developed a plan for disseminating study results, as well as a strategy for successful implementation of our research findings into practice.
